# Supplementary material for: Vapor-induced phase-separation-enabled versatile direct ink writing
Source: Nat Commun. 2024 Apr 9;15:3058. doi: 10.1038/s41467-024-47452-9 (PMC11003993; doi:10.1038/s41467-024-47452-9)
Supplement: Supplementary file 1 — Supplementary Information [file 41467_2024_47452_MOESM1_ESM.pdf]

Supplementary Information

Vapor-Induced Phase Separation-Enabled Versatile Direct Ink Writing

Marc Sole-Gras[1], Bing Ren[1], Benjamin J. Ryder[2], Jinqun Ge[3], Jinge Huang[4], Wenxuan Chai[1], Jun Yin[5], Gerhard E. Fuchs[2], Guoan Wang[3], Xiuping Jiang[4], Yong Huang[1,2,*]

[1]*Department of Mechanical and Aerospace Engineering, University of Florida, Gainesville, FL, USA*
[2]*Department of Materials Science and Engineering, University of Florida, Gainesville, FL, USA*
[3]*Department of Electrical Engineering, University of South Carolina, Columbia, SC, USA*
[4]*Department of Food, Nutrition, and Packaging Sciences, Clemson University, Clemson, SC, USA*
[5]*College of Mechanical Engineering, Zhejiang University, Hangzhou 310028, China*
∗Correspondence to yongh@ufl.edu

Contents

1. Supplementary Notes **1**
1.1 Solubility analysis 1
1.2 VIPS dynamics 3
1.2.1 Ternary phase diagrams 3
1.2.2 Solvent-polymer interaction 4
1.3 Evolution of the solidification front and effect of demixing rate on the filament diameter 6
1.4 Printability and print shape accuracy 8
1.5 Morphology and porosity of polymeric filaments 12
1.6 3D printing of overhang filaments in air 16
1.7 Thermogravimetric analysis 17
1.8 Sintering dynamics 18
1.8.1 Sintering cycle and conditions 18
1.8.2 316L powder composition 18
1.8.3 Metallographic studies and chemical composition of sintered parts 18
1.9 Densification results 20
1.9.1 Mechanical properties of dense parts 20
1.9.2 Post-processing feasibility 20
1.10 Horn antenna printing 22
1.11 Respirator composite printing 25
1.11.1 Printing 25
1.11.2 Antibacterial testing 25
1.12 316L-hydroxyapatite EDS readings 26
1.13 Porous structure printing 27
1.14 Mechanical properties and fractometry of porous parts 28
1.15 Cellular testing protocol and data 29
1.16 Scaffold biocompatibility evaluation 30
2. Supplementary References 31

1.1. Solubility analysis

The relative energy difference (RED) parameter[1] is used to evaluate the suitability of a given polymer candidate for VIPS (vapor-induced phase separation) printability from a dissolution perspective, which shall present a value lower than the unity (RED<1) if the polymer is to be fully dissolved in a given solvent. This RED value is determined based on Hansen's chemical affinity relationships[2] as defined by Eqs. (1) and (2).

$$R_a^2 = 4(\delta_{d2} - \delta_{d1})^2 + (\delta_{p2} - \delta_{p1})^2 + (\delta_{h2} - \delta_{h1})^2 \quad \textbf{(1)}$$

$$RED = \frac{R_a}{R_0} \quad \textbf{(2)}$$

where R_a is the distance in the Hansen space, δ_d is the energy from dispersion forces between molecules (in $MPa^{0.5}$), δ_p is the energy from dipolar intermolecular forces between molecules (in $MPa^{0.5}$), δ_h is the energy from hydrogen bonds between molecules (in $MPa^{0.5}$), and R_0 is the interaction radius. Based on the published energy values, the calculated RED values for polyacrylonitrile (PAN) and acrylonitrile butadiene styrene (ABS) are listed in Supplementary Table 1, proving that these two polymers are dissolvable in dimethyl sulfoxide (DMSO).

Supplementary Table 1. RED calculations.

**Component**	**δ_d**	**δ_p**	**δ_h**	**R_0**	**RED**
DMSO[2]	18.4	16.4	10.2		
PAN[2]	21.7	14.1	9.1	10.9	0.65
ABS[3]	17.6	8.6	6.4	10.9	0.81

1.2. VIPS dynamics

1.2.1 Ternary phase diagrams

The ternary diagrams for the polymer, non-solvent, and solvent of interest herein are constructed to elucidate the phase-separation dynamics of vapor-induced phase separation 3D printing (VIPS-3DP). The Flory-Huggins (F-H) model[4,5] has been extensively used as a thermodynamic model to describe the behavior of and interactions from ternary systems formed by polymer, non-solvent, and solvent mixtures during the phase inversion process. Specifically, the Flory-Huggins equation for the Gibbs free energy of mixing of two components (i and j)[6] is shown in Eq. (3) in its adapted form[7]:

$$\frac{\Delta G_M}{RT} = n_1 ln\varphi_1 + n_2 ln\varphi_2 + n_3 ln\varphi_3 + g_{12}(u_2)n_1\varphi_2 + g_{13}(\varphi_3)n_1\varphi_3 + g_{23}(\varphi_3)n_2\varphi_3 \quad \textbf{(3)}$$

where R and T are the gas constant and temperature, n_i is the relative molar volume, $u_2 = \varphi_2/(\varphi_1 + \varphi_2)$, φ_i is the volume fraction, and g_{ij} is the inter-polymer interaction function of the compounds i and j. A ternary system is usually formed by a non-solvent (1), a solvent (2), and a polymer (3), which is plotted as a triangle.

It is important to determine the equilibrium boundaries that a ternary system presents as they define the demixing threshold conditions and demixing types. The equilibrium points are the binodal curve, which is usually determined mathematically by equalizing the potential energy as $\Delta\mu_{i,A} = \Delta\mu_{i,B}$, where A and B are the polymer-rich and polymer-lean phases, respectively. However, for successful modeling, it is required to accurately describe the inter-compound interaction parameter g_{ij}, which is material-dependent and concentration-dependent[8]. Thus, the determination of the equilibrium binodal curve has been empirically identified by the use of the titration method and expanded to all domains by the use of the linearized cloud point (LCP) curve[9]. The titration method aims to empirically determine the first point under non-equilibrium conditions, that is, via the identification of the component concentrations under which the first turbid product is obtained[10,11]. Specifically, different polymer-solvent solutions were prepared due to the unequal increase of viscosity for each polymer. ABS and PAN mixtures were 10, 20, 30, and 40% (w/v), and 5, 10, 15, and 20% (w/v) in DMSO, respectively. Controlled amounts of deionized water, acting as non-solvent, were titrated against the polymer-solvent mixture at 25 °C until the first cloudy appearance showed up. Upon further stirring, additional non-solvent was titrated if the solution cleared out (metastable point); if the solution maintained its turbidity appearance, this was taken as a valid cloud point. The cloud points were later correlated using the LCP curve[9] using Eq. (4) that correlates the weight fraction $\emptyset$ of the non-solvent (1), solvent (2), and polymer (3), respectively:

$$ln\left(\frac{\emptyset_1}{\emptyset_3}\right) = b\ ln\left(\frac{\emptyset_2}{\emptyset_3}\right) + a \quad \textbf{(4)}$$

The obtained values of the LCP curves for both polymeric systems as well as the regression fitness value R^2 are summarized in Supplementary Table 2:

Supplementary Table 2. LCP curve and R^2 values.

**Polymer**	**a**	**b**	R^2
ABS	-3.4199	1.3855	99.6
PAN	-2.7305	1.4973	99.8

The relationships established by the LCP can be extrapolated beyond the measured points by iterating all possible weight fractions for all components, knowing that all weight fractions add to the unit ($\varnothing_1 + \varnothing_2 + \varnothing_3 = 1$), so the binodal curves for both polymeric systems are plotted for all the feasible domains as shown in Supplementary Fig. 1.

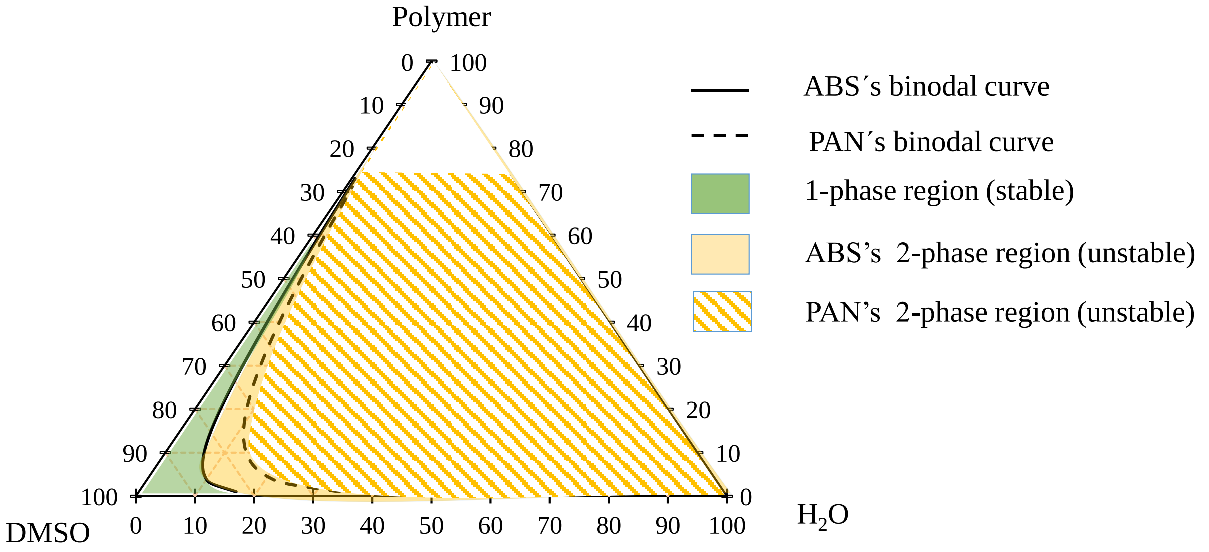

Supplementary Figure 1. Ternary diagrams for ABS and PAN-based ternary systems and their stable and unstable regions.

As seen from Supplementary Fig. 1, the stability region of the PAN system is greater than that of the ABS system, leading to the deposited material being able to absorb more water (higher non-solvent content) prior to demixing, that is, solidification. This fact explains the faster phase-separation dynamics of the ABS filaments that yield a higher achievable deposition speed as well as the RED value of ABS closer to unity as shown in Supplementary Table 1. This supports the conclusion that ABS has a weaker affinity with the solvent (DMSO) than the PAN counterpart, and thus, phase separating and demixing faster.

1.2.2 Solvent-polymer interaction

In this study, the kinetics of the polymeric VIPS are studied to enable the fabrication of polymer-based parts. Such kinetics, the resultant porosity, and the developed ternary system stability boundaries are shown to be heavily dependent on the material and printing conditions. It must be noted that such boundaries may not be exactly applicable to other evaporation or thermally-initiated phase-separation processes (air-casting or TIPS) as their thermodynamics are based on

notably different mechanisms, such as evaporation or heating. However, these stability boundaries may be applicable to other liquid-form non-solvent-induced phase separation (NIPS) mechanisms. Such stability boundaries are not affected by the significantly higher kinetics of the solvent-non-solvent exchange dynamics found in liquid-form NIPS, given that the amount of non-solvent in a given volume is more if found in the liquid-form process (NIPS) than its vapor-form process (VIPS). Nevertheless, the distinct exchange dynamics between the solvent and non-solvent are exemplified by the different non-solvent diffusion rates[12,13]. In that regard, delayed demixing is expected to occur using VIPS due to the lower solvent extraction rates when compared to NIPS, being the final porosity morphology also significantly affected[14].

Furthermore, the nature of the polymer chain and its composition affect the solubility in a desirable solvent, therefore affecting the printability of polymer-solvent-based inks using a VIPS-based printing process. This relationship is usually expressed using the interaction parameter between the polymer and solvent g_{23}, which can be usually determined by scattering, osmosis, or vapor-pressure methods[15].

Herein, the parameter g_{23} can be calculated using Hansen solubility parameters[2] as noted in Eq. (5):

$$\mathbf{g}_{ij} = \frac{\boldsymbol{v_i}}{RT}[(\boldsymbol{\delta}_{di} - \boldsymbol{\delta}_{dj})^{\mathbf{2}} + 0.25(\boldsymbol{\delta}_{pi} - \boldsymbol{\delta}_{pj})^{\mathbf{2}} + 0.25(\boldsymbol{\delta}_{hi} - \boldsymbol{\delta}_{hj})^{\mathbf{2}})] \quad \mathbf{(5)}$$

where i=2, j=3, v_i is the molar volume of component i, and R and T are the universal gas constant and absolute temperature. The energy values δ are listed in Supplementary Table 1, and the molar volume of DMSO is assumed 71.2 cm^3/g as noted by the manufacturer. By using Eq. (5), the interaction parameters g_{23} obtained for the ABS and PAN are approximately g_{23} (ABS) = 0.55 and g_{23} (PAN)= 0.35, respectively. Such differences in the polymer-solvent interaction parameters are the cause of the distinct behavior observed in the miscibility gap, as the g_{23} increase results in a narrower miscibility gap, and therefore, a reduced stability region[16-18].

1.3 Evolution of the solidification front and effect of demixing rate on the filament diameter

Understanding of solidification dynamics is of great importance for a successful VIPS-3DP, which is investigated by studying the evolution of the solidification front after filament printing. For such a solidification dynamics analysis, the solidification timescale is estimated via optical measurements, and Supplementary Fig. 2 presents an example, where it is shown a typical 20% (w/v) ABS-based ink being printed on a glass substrate and observed (bottom view) over time to quantify the solidification rate during the VIPS printing process by capturing the opacification of the filament. For the 20% (w/v) ink under 40% RH, it takes approximately 90 seconds for the opacification to be fully completed. The solidification front travels inwards (white arrows, inset). The printing speed needs to match the solidification rate for a good shape fidelity and proper inter-layer fusion. For the 20% (w/v) ink under higher RH, the opacification is a much more rapid process (<10 seconds as this is the time it takes for the contrast to adjust with our imaging setup). It should be pointed out that higher concentration ABS inks present significantly more opacity in the liquid phase, inhibiting any meaningful observation of the change in opacity over time.

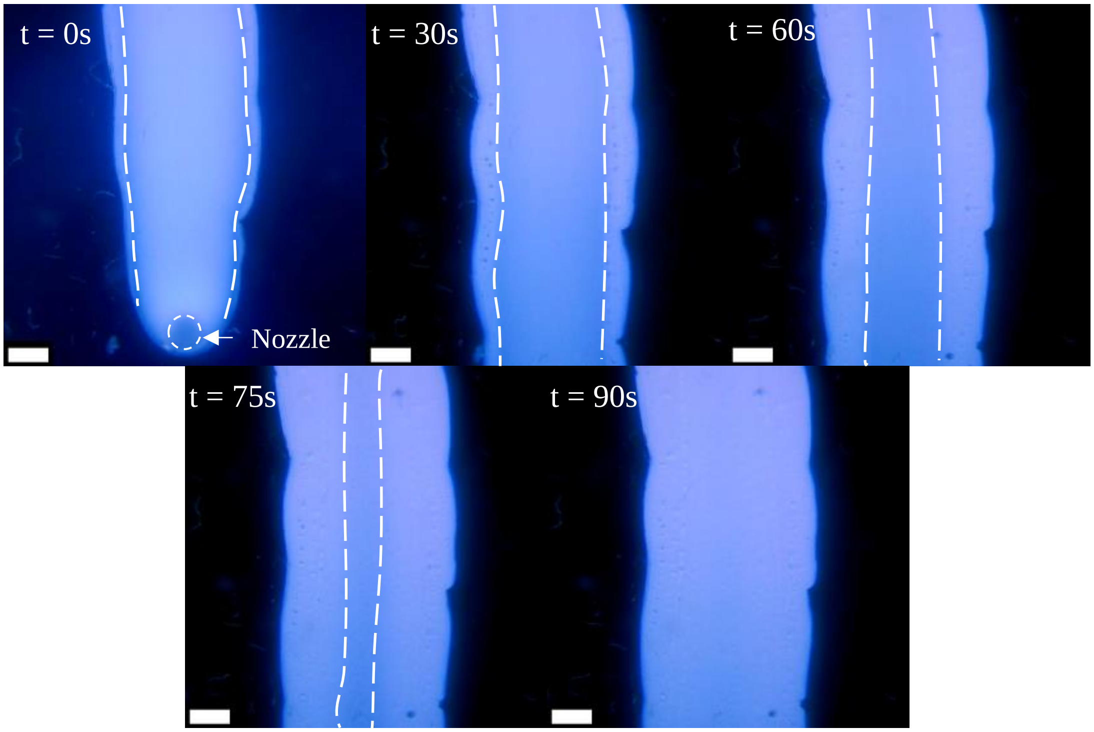

Supplementary Figure 2. Evolution of the solidification front of 20% (w/v) ABS ink at 40% RH over time showing 0, 30, 60, 75, and 90 seconds after filament printing. Scales bars: 2 mm.

During the VIPS process, the demixing rate is dependent on the polymer concentration and RH level, where a higher polymer concentration and a higher RH level lead to a higher demixing rate (Supplementary Fig. 3(a)). The solidification rate k ($k = \frac{Solidification\ front\ thickness\ (\mu m)}{Time\ (s)}$) increases from 0.02 µm/s to 0.26 µm/s for the 20% (w/v) ABS as the RH level increases from 40% to 99% (Supplementary Fig. 3(a-b)). A higher demixing rate allows the deposited ink to solidify faster and hold the shape promptly, while a lower demixing rate allows the ink to spread, leading to a flat shape with a larger diameter. As shown in Supplementary Fig. 3(b), the spreading of the

initially deposited filament leads to a larger diameter of a solidified filament at low RH levels (such as 40% and 70%), which is due to the slower demixing rate. At 99% RH, the diameter variation between the nozzle diameter and solidified filament significantly decreases, indicating less ink spreading and a better printing resolution.

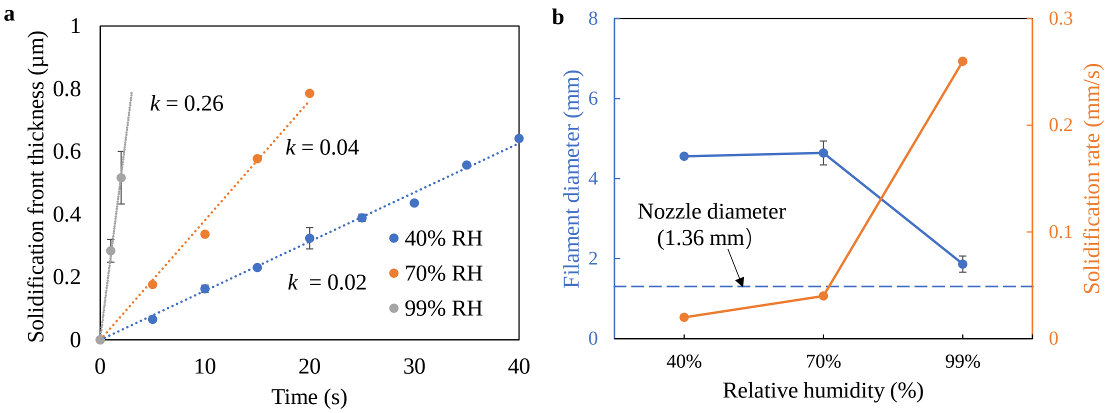

Supplementary Figure 3. Effects of relative humidity on solidification front thickness and filament diameter. (a) Evaluation of the solidification front of 20% (w/v) ABS ink under 40%, 70%, and 99% RH over time using a gauge 22 nozzle (diameter of 0.41 mm). (b) Effect of RH (40%-99%) on the printed 20% (w/v) ABS filament diameter using a gauge 15 nozzle (diameter of 1.36 mm) and a printing speed of 1.5 mm/s and effect of RH on the solidification rate *k*.

1.4 Printability and print shape accuracy

For a detailed printability study during VIPS-3DP, a printability factor ($Pr = \frac{L^2}{16A}$) is used to quantitatively evaluate the filament fidelity using a lattice design[19], shown in Supplementary Fig. 4(a) and (b), where L is the perimeter length of a single cell of the lattice and A is the measured blank area enclosed by the printed strands. *Pr* of 1 is the ideal value. *Pr* of less than 1 results in a smaller perimeter to area ratio than that is present in a square geometry. A value greater than 1 also indicates deformed filaments with an increased cell perimeter. Images of printed lattice structures of 20%-30% (w/v) ABS under 40% RH (0.10 mm nozzle at 5.0 mm/s) are shown in Supplementary Fig. 4(c). It shows that the 30% (w/v) ABS ink has the best shape fidelity, while 20% (w/v) ABS filaments spread and fuse together due to the low viscosity and slow solidification. The measured *Pr* values are present in Supplementary Fig. 4(d), which are consistent with the print images. The *Pr* value is also affected by the RH conditions as seen from Supplementary Fig. 4(e) and (f). For the 25% (w/v) ABS ink being printed at 3.0 mm/s using a 0.10 mm nozzle, the 70% RH condition results in the best printability factor around 1. The ink cannot have a good printability factor under 40% RH even when the printing speed is reduced from 5.0 mm/s to 3.0 mm/s in order to let the ink have sufficient time to solidify and retain a good shape fidelity. Even though printing under RH values of 99% may theoretically yield *Pr* values close to 1, it must be noted that such conditions are not advisable as the ink may solidify too fast and clog and block the dispensing nozzle, resulting in deformed filaments.

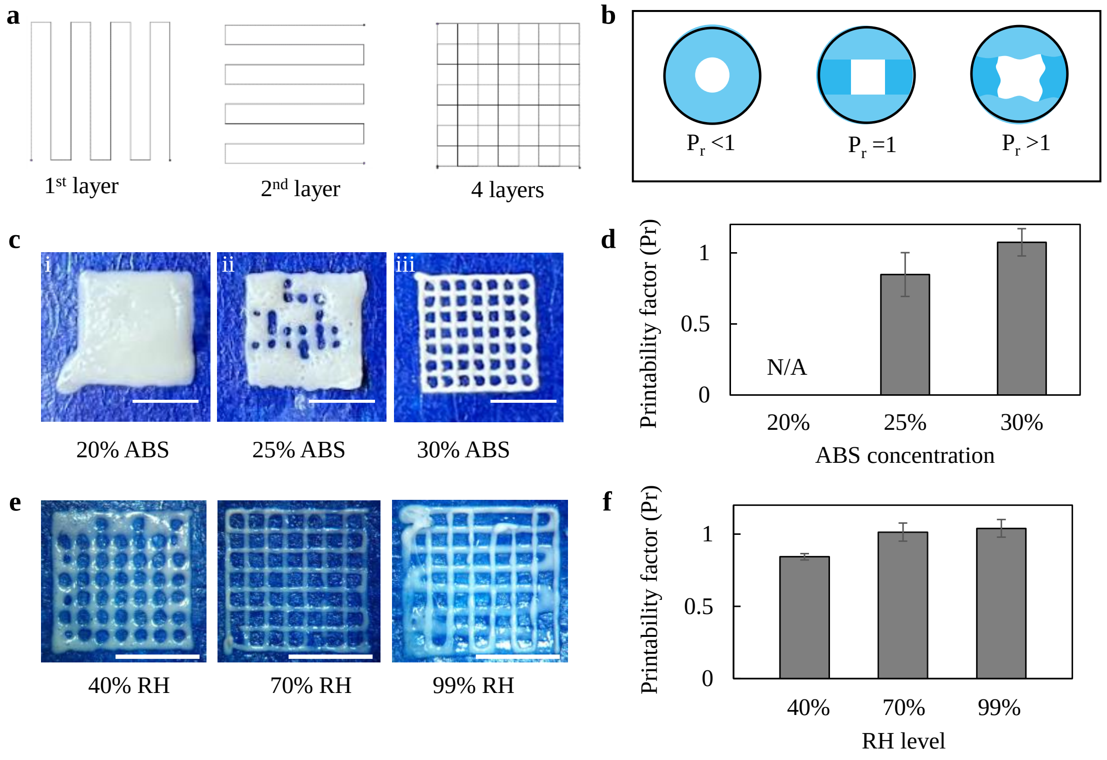

Supplementary Figure 4. Quantitative evaluation of printing fidelity. (**a**) Lattice design for printing evaluation. (**b**) Schematic of printability factor (*Pr*) measurement. (**c**) Printing results of 20-30% (w/v) ABS inks under 40% RH using a 0.10 mm nozzle at 5.0 mm/s. (**d**) *Pr* results for (**c**). (**e**) Printing results of 25% ABS ink under 40%, 70%, and 99% RH using a 0.10 mm nozzle at 3.0 mm/s. (**f**) *Pr* results for (**e**) (scale bars: 5 mm, and N/A: not applicable).

In terms of the print shape accuracy, resulting polymer parts may experience some post-printing shrinkage, given that solvent is extracted out of the deposited filament as a result of the phase separation process. The level of shrinkage depends on the RH value (Supplementary Fig. 5(a) and (b)), polymer concentration (Supplementary Fig. 5(a) and (c)), and polymer material (Supplementary Fig. 6). To study the effect of RH on the shape accuracy, a circular structure with an inner diameter (ID) of 10.0 mm, an out diameter (OD) of 10.9 mm, and a wall thickness of 0.45 mm (Supplementary Fig. 5(a)) is designed and printed to evaluate the print shape accuracy using a gauge 22 nozzle (diameter of 0.41 mm) at 4.0 mm/s. The 40% (w/v) ABS ink was printed under the 40%, 70%, and 99% RH levels, and the printing results are shown in Supplementary Fig. 5(b). The printed circles are smooth with good shape fidelity, and the measured ID, OD, and wall thickness are close to the design values (Supplementary Fig. 5(d)). Of them, the 40% RH level results in the best print shape accuracy with slight shrinkage.

To study the effect of polymer concentration on the shape accuracy, a circular structure was similarly printed while using the 20%, 30%, and 40% (w/v) ABS inks using a gauge 22 nozzle (diameter of 0.41 mm) at 4.0 mm/s under 40% RH (Supplementary Fig. 5(c)). The 20% ABS ink has severe spreading due to the low polymeric concentration, resulting in a higher OD and a lower ID than design values, while the 30% and 40% (w/v) ABS inks result in relatively accurate prints due to the combined effect of ink viscosity (for spreading) and solidification speed (Supplementary Fig. 5(e)). Of them, the 40% (w/v) ABS ink results in the best print shape accuracy with slight deviation.

As observed, the good combination of RH and polymer concentration is 70% RH and 30-40% (w/v) ABS during VIPS-3DP ABS printing.

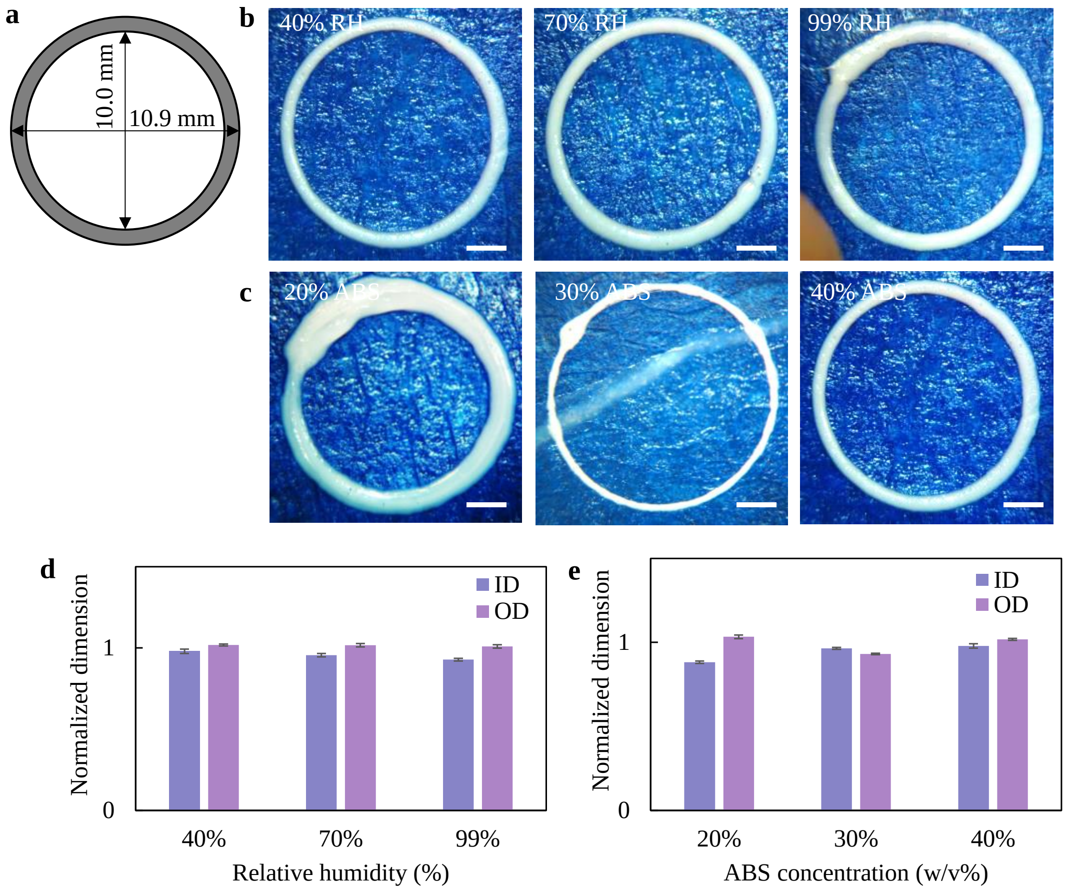

Supplementary Figure 5. Post-printing solidification-induced filament diameter variation as a function of RH and polymer concentration. (**a**) Shape design, (**b**) printing results of 40% (w/v) ABS under 40%, 70%, and 99% RH levels, (**c**) printing results of 20%, 30%, and 40% (w/v) ABS under 40% RH, and (**d-e**) measurements of the inner diameter (ID) and out diameter (OD) of (**b-c**), normalized to the design values (dashed lines). (Scale bars: 2 mm)

To study the effect of polymer material on the shape accuracy, both ABS and polyacrylonitrile (PAN) were used as two example polymer materials with different chemical affinities with the solvent (DMSO). Because PAN has a better affinity due to a lower relative energy difference (RED) value than ABS (Supplementary Table 1), it leads to a slower phase-separation process. The slow solidification rate of PAN allows it to shrink as it gets slowly solidified and thus, presenting a high post-printing shrinkage degree and smaller pore sizes. In contrast, ABS solidifies fast due to the rapid solvent extraction, having a low post-printing shrinkage degree. An example of the as-printed and as-solidified parts, 20% (w/v) PAN-based semicircles before and after solidification are shown in Supplementary Fig. 6(a) and (b), presenting a clear reduction in diameter as indicated by black arrows. For comparison in terms of the print shape accuracy, the shrinkage magnitude of the 20% (w/v) ABS and 20% (w/v) PAN inks is quantified to be approximately 3% and 30%, respectively, as seen in Supplementary Fig. 6(b), showing that polymers having a low RED value may shrink more significantly.

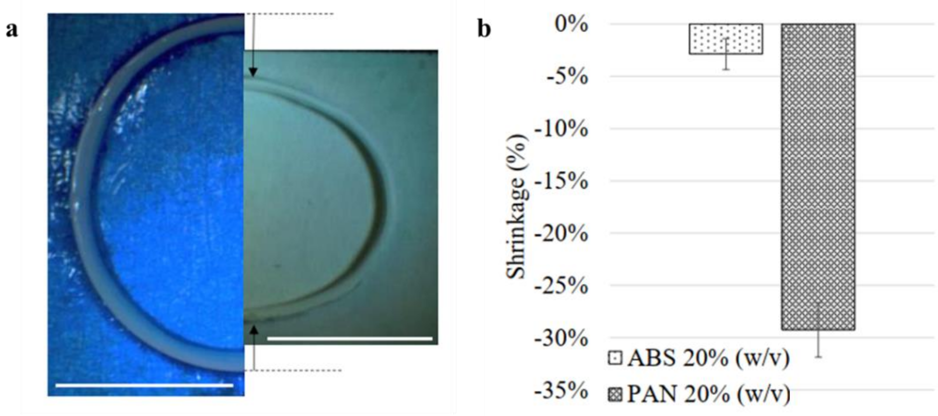

Supplementary Figure 6. Shrinkage characterization. (**a**) Tube printing using 20% (w/v) PAN (scale bar = 10 mm) and (**b**) shrinkage measurements for ABS and PAN.

1.5 Morphology and porosity of polymeric filaments

The printed filaments present a dense skin in the outermost layer due to being the first surface to have water-induced phase separation. Such dense skin layer with low porosity has been reported in similar VIPS-based solidification cases[20,21]. Generally speaking, the complete formation of dense skin layers during VIPS 3DP is largely dependent on the phase-separation kinetics of a polymer solution. Faster phase-separation kinetics promote the formation of hardening skin layer that leads to consecutively deposited layers not being fused well due to the significantly different hardening degree of their contact surfaces. If a previously deposited layer has a notable solvent-extraction time in the outer surface layer under fast phase-separation kinetics, it may harden well and form a dense skin layer before a subsequent filament is deposited. The difference in hardening status and the presence of a thin layer of solvent and non-solvent may result in insufficient bonding between two consecutive layers and a visible interface (Supplementary Fig. 7(a)) using a scanning electron microscope (SEM, Hitachi S3000, Hitachi High-Technologies Co., Tokyo, Japan). This phenomenon is particularly pronounced when ABS is used.

In addition, faster phase-separation dynamics play an effect on the inner porosity morphology as the presence of macro- and micro-voids can be found in the innermost layers, which are the last ones to demix and solidify[21] (Supplementary Fig. 7(a), inset). On the other hand, if the phase-separation kinetics are slow, the solvent/non-solvent exchange-related mass transfer may be reflected as a reduction of the filament cross section[22] given the lower hardening degree of the filament, leading to well-bonded filaments (Supplementary Fig. 7(b)) and unnoticeable pores at the micron level being present in the filament cross section (Supplementary Fig. 7(b), inset). This is the case when PAN is used.

From a morphological perspective, distinct porous morphologies can be observed during ABS and PAN printing. ABS does present a clear porous morphology (Supplementary Fig. 8(a)) where the pores are in the range of the hundreds of microns in both the intermediate and bottom regions (Supplementary Fig. 8(b)-(c)). The PAN counterparts do not show any obvious and observable pores in the micron range (Supplementary Fig. 8(d)). Further mercury intrusion porosimetry (Poremaster, Quantachrome, Anton Paar, Graz, Austria) reveals that the PAN-based structures are porous in nature whose pore size averages 140 nm in diameter (Supplementary Fig. 8(e)). Interestingly, the symmetry of the porosity observed for the fast-separating polymer in Supplementary Fig. 8(a) is an adaptation of the reported one-dimensional (1D) asymmetry of the thin membranes obtained via VIPS[23].

If the ABS concentration increases to 40% (w/v), the asymmetric porosity morphology resembles the one reported elsewhere[24] since the phase-separation kinetics are higher due to the less amount of solvent to be extracted, and the results are shown in Supplementary Fig. 9. The printed filaments present a dense skin (Supplementary Fig. 9(a)) in the outermost layer due to being the first surface to be in contact with the water mist, which acted as a non-solvent. As such, this dense skin layer with low porosity is expected to be found in all cases[20,21], and such a morphology is observed in 20, 40, and 60% (w/v) ABS. The intermediate region of the filament presents a cell-like pore (Supplementary Fig. 9(b)). The presence of macro- and micro-voids can be found in the innermost layers, which are the last ones to demix[21] (Supplementary Fig. 9(c)) that are in the range of hundreds of microns.

As seen in Supplementary Fig. 10, for the selected ABS concentrations (40% and 60%, typical concentrations used for polymer printing in this research) and RH conditions (40%, 70%, and 99%) investigated, the overall porosity varies between 35% and 45%, and there is no significant difference

observed among these samples (Supplementary Fig. 10 (a)). Most of voids found on the cross section of whole filaments present a pore size between 1 and 5 μm (Supplementary Fig. 10 (b-c)). Although no change in the overall porosity is observed as the RH level increases, the average pore size decreases as observed in the distribution plots (Supplementary Fig. 10 (b) (from 2.78 to 2.59 to 2.34 μm) and (c) (from 2.81 to 2.50 to 1.87 μm)) wherein the smaller voids tend to have a higher frequency. This phenomenon is observed for both 40% and 60% (w/v) ABS, and the decrease in the pore size is more pronounced with the 60% (w/v) ABS, meaning that higher concentration polymers such as ABS may be more prone to form smaller voids under higher RH levels.

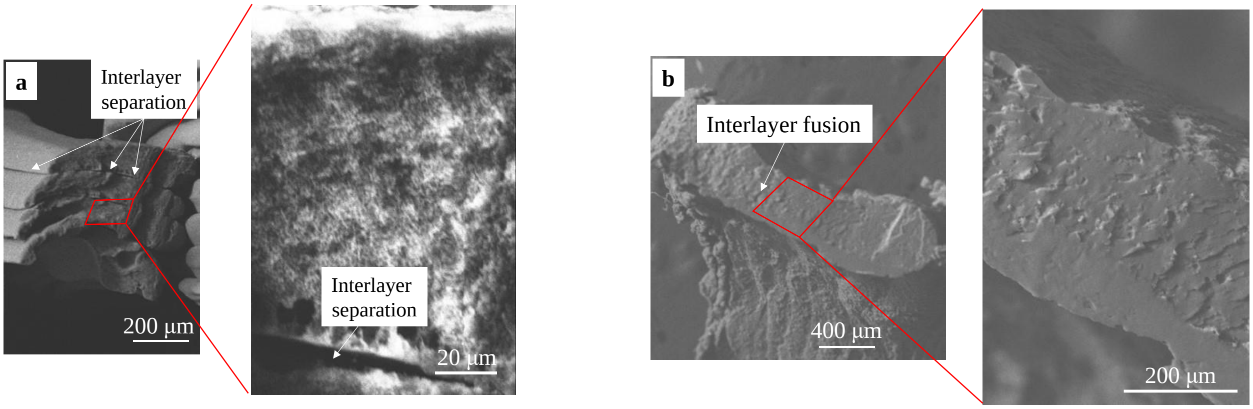

Supplementary Figure 7. (**a**) SEM images of 20% (w/v) ABS, (**b**) SEM images of 20% (w/v) PAN.

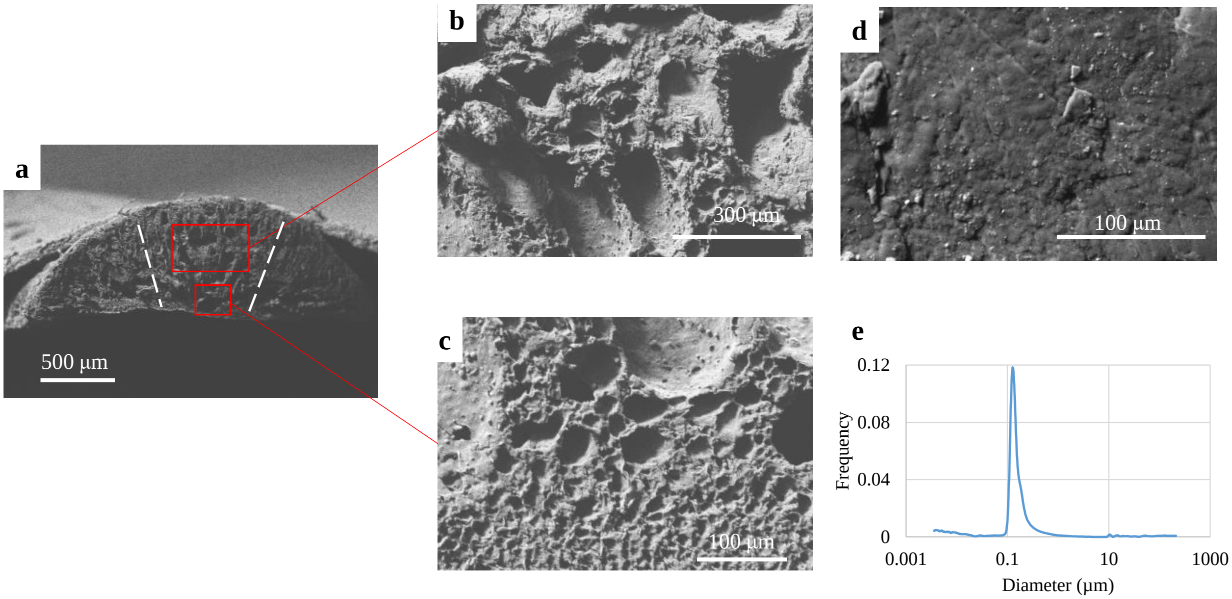

Supplementary Figure 8. Representative images of filament morphology (semi-circle herein). (**a**) Morphology of a 20% (w/v) ABS filament, and magnifications of intermediate (**b**) and bottom (**c**) regions. (**d**) Magnified morphology of a 20% (w/v) PAN filament and (**e**) pore size distribution obtained using mercury intrusion porosimetry.

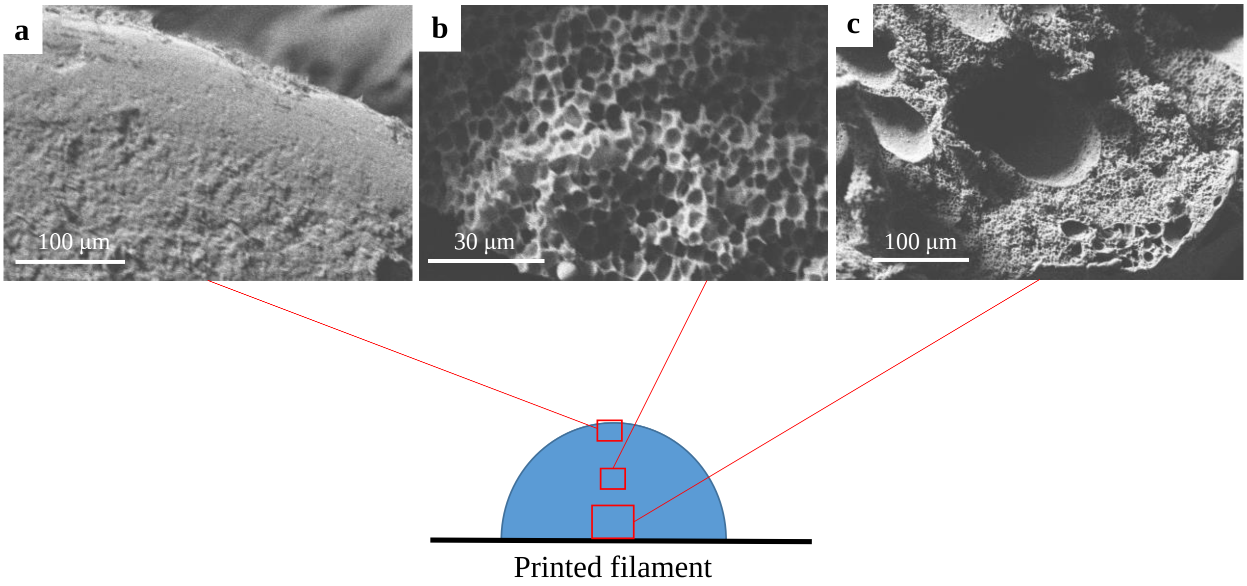

Supplementary Figure 9. Detailed view of the porosity morphology of filament. Magnified views of top (**a**), intermediate (**b**), and bottom (**c**) regions of a 40% (w/v) ABS-based filament.

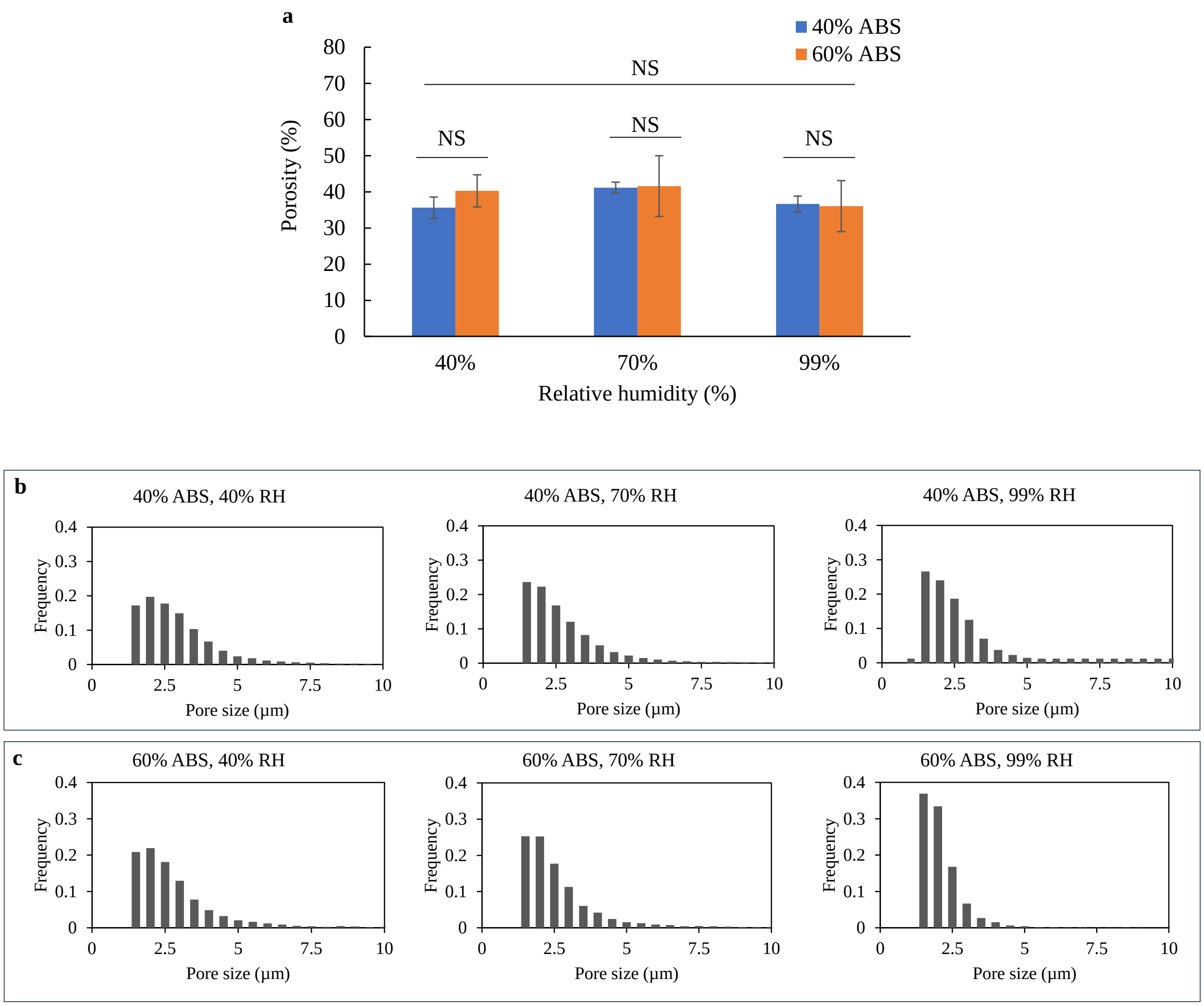

Supplementary Figure 10. Porosity characterization. (a) Porosity of 40% and 60% (w/v) ABS, and pore size distribution of (b) 40% and (c) 60% (w/v) ABS under different RH levels (40%, 70%, and 99%) using a 0.41 mm nozzle at 1.0 mm/s. ("NS" indicates not significant ($p > 0.05$) in the porosity value between the samples (paired *t* test).)

1.6 3D printing of overhang filaments in air

Successful deposition of completely overhanging continuous polymer-based filaments in air is achieved by using 40% (w/v) ABS under the nebulized condition, with a nozzle speed of 1.0 mm/s. The printing conditions were fixed, so the resulting filament height was approximately 1 mm. The deposited filaments were approximately 1 mm in diameter and could withstand a maximum span of 35 mm without noticeable gravity-induced deflection as seen from Supplementary Movie 1. Solutions with lower polymeric concentrations and/or slower speeds resulted in discontinuities in the deposited line or significant filament deflection.

1.7 Thermogravimetric analysis

The dynamics of debinding process of green parts, resulting in the polymer binder being volatilized, are studied herein. The debinding performances of ABS and PAN are investigated using thermogravimetric analysis (TGA, Q5000, TA Instruments) to assess the mass loss during various thermal cycles using 5 mg samples. The TGA study was conducted with nitrogen flowing at 60 mL/min and heating rates of 5 and 10 °C/min. The TGA curves of ABS and PAN are shown in Supplementary Fig. 11(a) and (b). ABS rapidly decomposes in a single step from 345°C, and the polymeric mass decays to a remainder of 3% at 420°C and has no significant amount remaining (<1%) at 470°C. PAN decomposes on a significantly different behavior: no mass loss is observed until the point of 270°C. From that temperature point, three decomposition rates are observed: a rapid mass loss rate in the 270-300°C region, a moderate mass loss rate in the 300-470°C, and a low mass loss rate occurring from 470 to 1000°C. It is noted that PAN samples still present a remainder of 20% of mass at the highest operative temperature of 1000°C. The high withstanding temperature of PAN is the main reason for it being used as a precursor of carbon fibers given its high non-volatility at high temperatures, and this property is due to its cyclized structure and high-carbon content in 1000-1300°C[25,26]. Thus, the use of PAN as a binder implies the presence of high carbon contents that may impact its suitability to bind metals due to undesired carbide formation. As such, ABS is considered a better candidate to act as a binder for VIPS-3DP purposes, in particular, for metal printing, given that its complete polymer burnout occurs in the 420-470°C range.

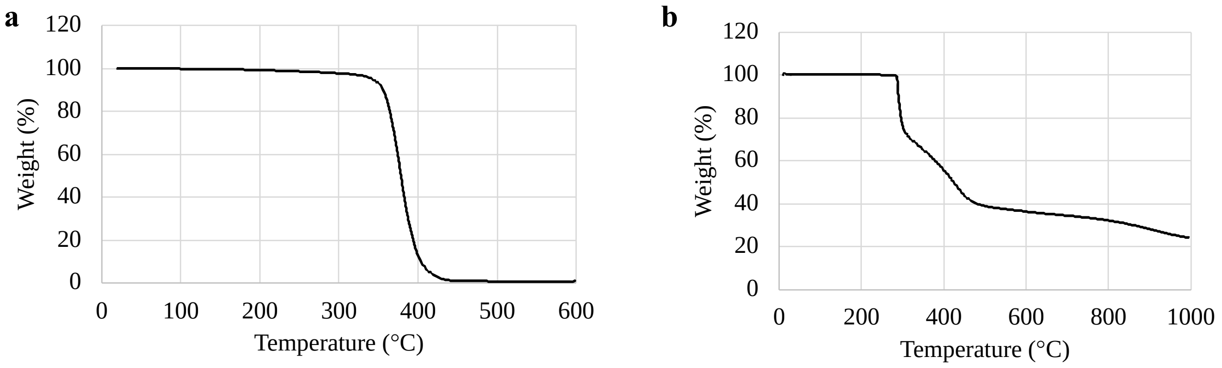

Supplementary Figure 11. TGA curves of (**a**) ABS and (**b**) PAN.

1.8 Sintering dynamics

1.8.1 Sintering cycle and conditions

The sintering cycle is detailed as follows: the heating and cooling rates were 5°C/min; a dwell time of 30 minutes at 300°C was used to avoid thermal cracking on the alumina tube; the debinding cycle was based on the TGA data and set at 500°C for 60 min; and the sintering temperature was set at 1300°C for 2 hours, where solid-state sintering of the 316L-based powders was induced under vacuum conditions. The temperature profile of the furnace is shown in Supplementary Fig. 12.

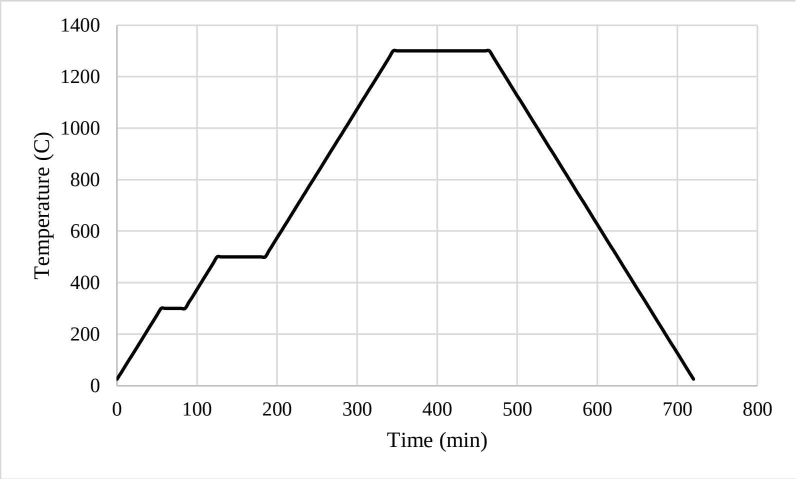

Supplementary Figure 12. Thermal profile for debinding and sintering of ABS-316L-based printed parts.

1.8.2 316L powder composition

The chemical composition of the 316L powders (D_{10}: 4.61 µm, D_{50}: 12.58 µm, D_{90}: 24.44 µm, MP0100, MSE Supplies, Tucson, AZ, USA) used herein is listed in Supplementary Table 3.

Supplementary Table 3. 316L powder composition.

**Element**	**Wt%**
Cr	16.95
Ni	10.84
Mo	2.36
Mn	0.73
Si	0.29
P	0.01
C	0.007
S	0.003
Fe	balance

1.8.3 Metallographic studies and chemical composition of sintered parts

Further characterization of the printed 316L parts was carried out to determine their final composition. The printed parts were cut using a diamond saw, mounted in epoxy resin, and

polished using silicon carbide diamond pads using water as a lubricant. The polished surfaces were analyzed using energy-dispersive spectroscopy (EDS, Hitachi S3000, Hitachi High-Technologies Co., Tokyo, Japan). As shown in Supplementary Fig. 13(a), the use of ABS results in an approximately 8% porosity present, and the pores are located along the grain boundaries. The pore size is on the order of 4 μm. Interestingly, PAN-based samples do not have such pores (Supplementary Fig. 13(b)), which may be due to the significant shrinkage of the polymeric phase during printing (Supplementary Fig. 6(b)). From a composition perspective, the EDS results confirm the presence of the same elements as the initial composition of the 316L powders if ABS is used as a binder due to its easiness of volatilization (Supplementary Fig. 13(c)), and there is no noticeable residue from the binder composition. Differently, the PAN-based sintered parts do show a significant presence of carbon (>30%) (Supplementary Fig. 13(d)), which is due to the high-carbon content of the PAN structure in the temperature range of 1000 – 1300°C[25,26].

It is noted that under the current vacuum-based sintering, no noticeable loss of chromium is observed as expected due to the high vapor pressure of chromium at elevated temperatures[27]. This is caused by the current vacuum conditions not meeting the extremely high vacuum level of industrial powder metallurgy routes.

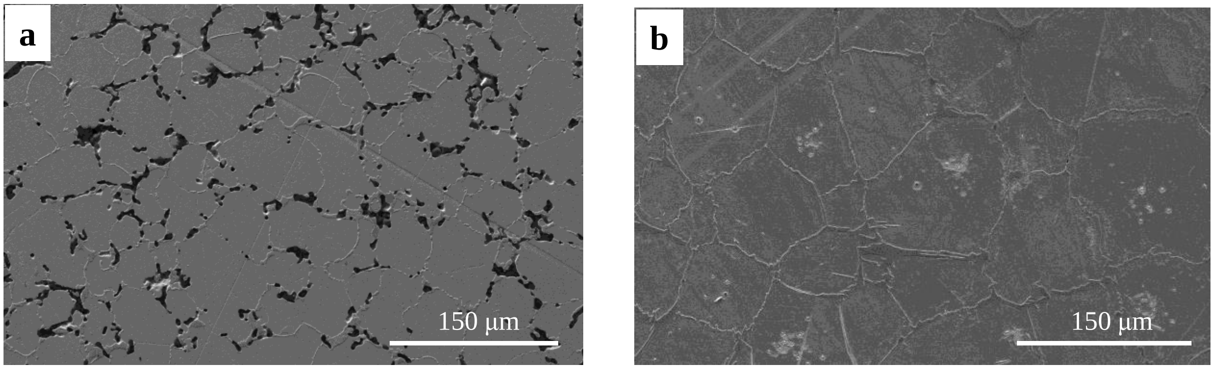

c

Element	Weight %	Atomic %	Net Int.	Error %	Kratio	Z	A	F
CrK	18.33	19.52	1183.39	3.50	0.2193	1.0008	0.9891	1.2086
FeK	71.60	70.99	2963.79	2.39	0.6979	0.9981	0.9641	1.0129
NiK	10.07	9.50	301.24	5.55	0.0913	1.0114	0.8915	1.0052

d

Element	Weight %	Atomic %	Net Int.	Error %	Kratio	Z	A	F
C K	35.47	71.28	1306.02	9.99	0.1271	1.1817	0.3031	1.0000
AlK	1.65	1.47	208.46	10.61	0.0081	1.0173	0.4799	1.0045
MoL	6.48	1.63	552.31	3.53	0.0522	0.7976	1.0033	1.0068
CrK	38.52	17.88	3306.44	2.26	0.3547	0.8889	0.9994	1.0363
MnK	1.58	0.69	115.35	19.29	0.0139	0.8707	0.9947	1.0160
FeK	16.30	7.05	996.84	3.84	0.1359	0.8851	0.9330	1.0090

Supplementary Figure 13. SEM images and EDS readings of cross sections of ABS- and PAN-based parts after polishing and etching. (**a**) ABS part and (**b**) PAN part SEM images. EDS readings of (**c**) ABS-based and (**d**) PAN-based parts.

1.9 Densification results

1.9.1 Mechanical properties of dense parts

Flat stainless-steel dog-bone specimens were printed using different material conditions, and their dimensions were approximately 30-40 mm long and 8-12 mm wide after sintering. Various mechanical properties such as ultimate tensile strength (UTS), were measured (6800 Series, Instron, Norwood, MA, USA) using a crosshead speed of 6-7 mm/min (ASTM E8-03). Three independent trials were conducted under the same conditions. The minor differences in UTS values are attributed to the presence of porosity, microcracks at the surface of the powders, impurities, and segregation[28,29] when compared to those made using the other fabrication routes.

1.9.2 Post-processing feasibility

To demonstrate the ability of the printed parts to undergo regular post-processing machining operations, some stainless-steel parts were sandblasted and tap threaded. Supplementary Fig. 14(a) presents a different view of the sandblasted part in addition to those shown in Fig. 3. The sand-blasting operation was conducted using a sand blaster (Blast It All, Model #3636-7DC, Salisbury, NC), which successfully removed the outer layer of the sintered part formed by the debinding and sintering debris, presenting a smooth and shining surface without compromising the integrity of the parts that include thin blade-like features. The near-theoretical density of the steel allows the thin features to withstand the sandblasting process as shown by the lack of deformation of the thin blades.

Moreover, a tap threading process was applied to a 3D printed and sintered zig-zag tube (Supplementary Fig. 14(b)), where a thread of 1/8 NPT was added to one end of the tube (Supplementary Fig. 14(c)). A push-to-connect fitting was assembled using the tap thread process, and no liquid leak was observed when blue-colored water flew through (Supplementary Movie 2).

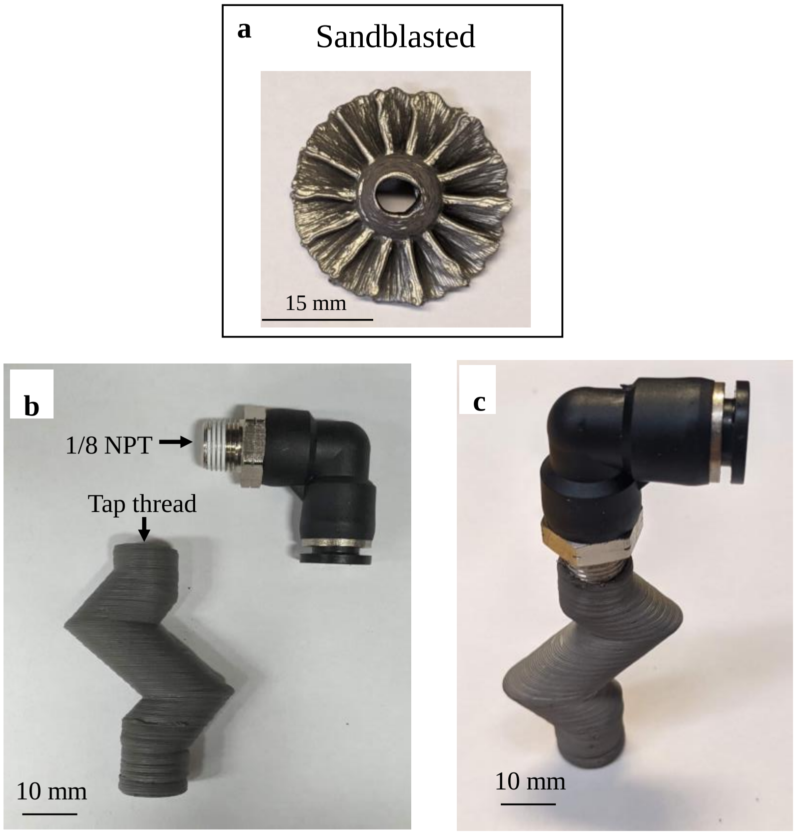

Supplementary Figure 14. (**a**) A different view of a sandblasted turbine-like part. (**b**) Tap threading summary and (**c**) tube and connector assembly.

1.10 Horn antenna printing

With the development of modern wireless communications, wider and higher frequency bands are employed to provide more bandwidths for high data-rate transfer, which results in the requirement of more complex and smaller antennas[30]. Generally speaking, the behavior of working antennas is based on the assembly of various sets of parts, which include the parabolic reflector, feeding guidelines, and supports for stability purposes. Therefore, the manufacturing route of such antennas requires specialized labor and may present high costs and time-consuming assembling steps. Thus, an emerging trend of designing antennas from traditional two-dimensional (2D) to 3D has gained momentum in recent years, as it provides more design freedoms, improves space utilization efficiency, simplifies manufacturing routes, and reduces manufacturing costs. As such, 3D printing has been pioneered as a powerful tool for antenna manufacturing that easily adapts to the variability of required designs in a cost-effective manner.

Herein the design of the fabricated horn antenna and its measured results are discussed. The horn antenna is known as a radiating element that has the shape of a horn. It can be considered as a radio frequency (RF) transformer or impedance match between the waveguide feeder and free space, which has an impedance of 377 Ω. The behavior and efficiency of the horn antennas are based on their geometry, specifically on the dimensions of the opening to be able to absorb the electric and magnetic field radiations, which are named the E-plane and H-plane, respectively[31]. For instance, the general parameters to consider in the design phase for a horn antenna are shown in Supplementary Fig. 15. For a desired operating frequency of a horn antenna, the standard waveguide with specific dimensions of a and b can be found elsewhere[32]. The designed horn antenna has a rectangle waveguide with the dimensions of a and b (Supplementary Fig. 15(a)), whereas the a_1 and b_1 variables denote the side lengths of the horn end at the E-plane and H-plane, respectively. Supplementary Fig. 15(b) represents the antenna geometry in the E-plane, where the R_e variable represents the distance between the top and bottom horn apertures. The horn in the E plane is determined by the diameter of ρ_1 and ρ_e. Similarly, the horn in the H-plane is defined by the parameter of R_h, ρ_2, and ρ_h as illustrated in Supplementary Fig. 15(c). A SubMiniature version A (SMA) connector is employed to transfer the signal from the coaxial cable to the rectangle waveguide.

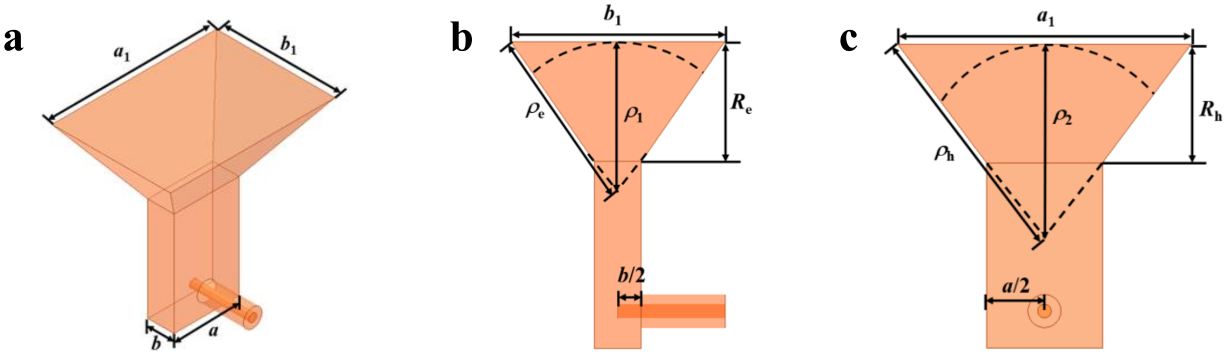

Supplementary Figure 15. Geometrical configuration of horn antenna. (**a**) 3D view. (**b**) E-plane. (**c**) H-plane.

The gain G of such a horn antenna is given in Eq. (6):

$$G = \frac{4\pi}{\lambda^2}\varepsilon_{ap}a_1b_1 \quad \textbf{(6)}$$

where $\mathcal{E}_{ap}$ is the aperture efficiency and λ is the wavelength in free space. The ε_{ap} value is typically chosen as 0.511 for an optimum horn antenna[33]. To achieve a given gain, the dimensions of the horn are determined Eqs. (7)-(11):

$$a_1^4 - aa_1^3 + \frac{3bG\lambda^2}{8\pi\varepsilon_{ap}}a_1 = \frac{3G^2\lambda^4}{32\pi^2\varepsilon_{ap}^2} \quad \textbf{(7)}$$

$$R_e = R_h = \frac{a_1-a}{3\lambda}a_1 \quad \textbf{(8)}$$

$$b_1 = \frac{1}{2}(b + \sqrt{b^2 + 8\lambda R_e}) \quad \textbf{(9)}$$

$$\frac{\rho_1}{R_e} = \frac{b_1}{b_1-b} \quad \textbf{(10)}$$

$$\frac{\rho_2}{R_h} = \frac{a_1}{a_1-a} \quad \textbf{(11)}$$

To demonstrate the suitability of 3D printing for antenna manufacturing, a horn antenna working at 22 GHz with a gain of 12 dB is designed as an example. Based on the previous formulas, a K-band standard rectangular waveguide *(a* = 10.7 mm, *b* = 4.3 mm) is used, and the dimensions of the horn are calculated as: a_1 = 27.1 mm, b_1 = 19.5 mm, R_e = R_h = 10.9 mm, ρ_1 = 13.9 mm, ρ_2 = 18.0 mm, ρ_e =17.0 mm, and ρ_h = 22.5 mm. As depicted in Supplementary Fig. 16(a), the lowest return loss of -16.5 dB is simulated at 22.7 GHz by Ansys HFSS. Further improvement of the design is carried out in order to achieve the best performance at 22 GHz, and the antenna dimensions are optimized to closely mimic the geometries of the 3D printed antenna: a_1 = 28.2 mm, b_1 = 20.4 mm, R_e = R_h = 11.5 mm, ρ_1 = 14.6 mm, ρ_2 = 18.6 mm, ρ_e =17.8 mm, and ρ_h = 23.3 mm. It can be observed from Supplementary Fig. 16(b) the 3D radiation pattern of the optimized horn antenna at its operating frequency, where the max gain of 14.2 dB is found. The modeled results of both original and optimized designs are shown in Supplementary Fig. 16(a), where the solid line depicts that the optimized antenna realizes the best radiation at 22 GHz with a reflection coefficient (S_{11}) of -16.13 dB.

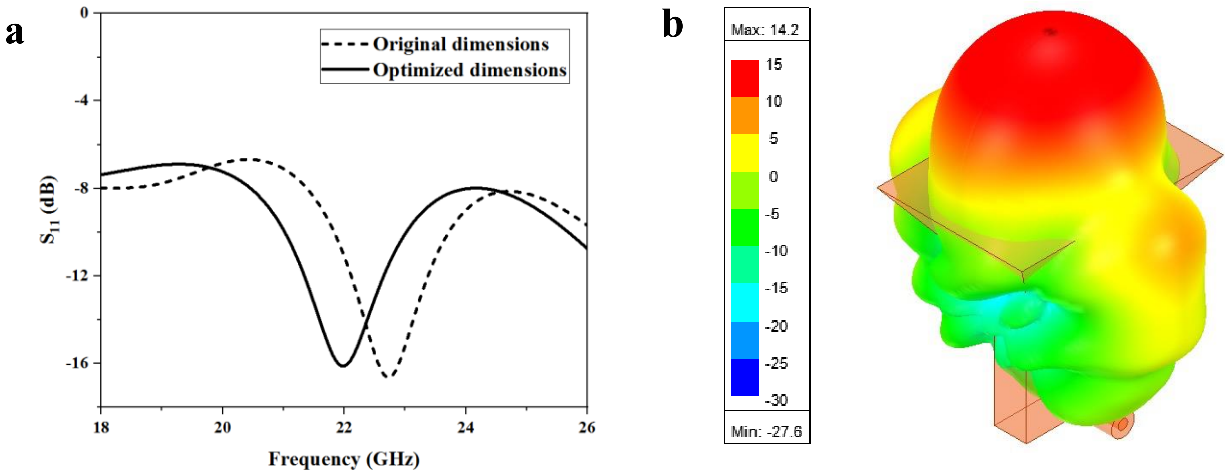

Supplementary Figure 16. Simulated results. (**a**) Return loss and (**b**) 3D radiation pattern at 22 GHz.

Furthermore, a steel-based horn antenna with the optimized dimensions was fabricated by VIPS-3DP (Supplementary Fig. 17). Due to the limitations of the sintering chamber, its final geometry is reduced as a_1 = 21.5 mm, b_1 = 14.9 mm, R_e = R_h = 9.5 mm, ρ_1 = 13.6 mm, ρ_2 = 12.6 mm, ρ_e =15.5 mm, and ρ_h = 16.6 mm. The change of the dimensions notably modifies the irradiation performance, as the printed antenna has a -10 dB bandwidth of 4.4 GHz, and the lowest reflection coefficient (S_{11}) is measured as -36.58 dB at 24.5 GHz (Fig. 3(o) in the manuscript, solid line) using a vector network analyzer (ZVA67, Rohde & Schwarz, Germany). This is higher than the ideal working frequency of 22 GHz, which may be due to minor dimensional differences. Further simulations based on the printed dimensions yield the modeling results (Fig. 3(o) in the manuscript, dashed line) matching these obtained experimentally. While an acceptable behavioral agreement between the measured and simulated results is observed, the small noise-based difference in the magnitude of the reflection coefficient S_{11} may be caused by two factors: the inner surface of the horn antenna presenting a step-wise morphology due to the hardening of adjacent filaments and the measurement-induced noise. In conclusion, the fabricated antenna presents good performance from 22.3 GHz to 26.7 GHz, which demonstrates the successful application of the VIPS-3DP technology for RF uses.

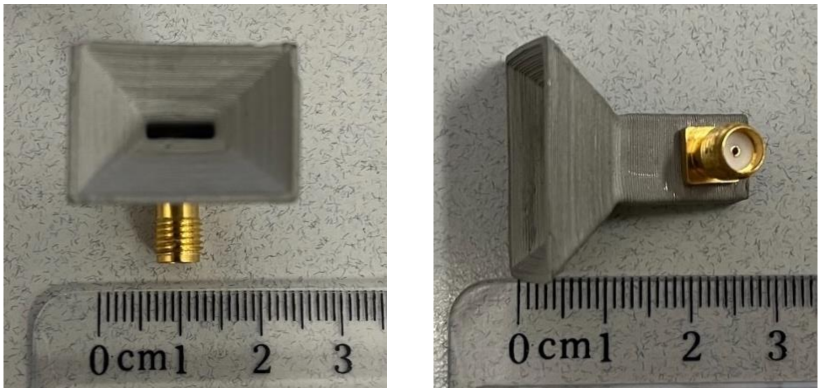

Supplementary Figure 17. Photographs of the 3D printed horn antenna.

1.11 Respirator composite printing

1.11.1 Printing

The preparation of the printing material, mixing the antiviral particles with a dissolved polymer solution and obtaining a homogeneous ink suspension for 3D printing, involves three steps. The first step consists of mixing the polymeric inks separately: filaments of acrylonitrile butadiene styrene (ABS, Stratasys, MI, USA) and thermoplastic polyurethane (TPU, Ellastollan, BASF, Germany) are mixed with the dimethyl sulfoxide solvent (DMSO, Fisher, NJ, USA) with concentrations of 40% and 20% (w/v), respectively. From a polymeric view, a mixture of dissolved ABS and thermoplastic polyurethane (TPU) is chosen due to the desired balance between the stiffness and elasticity brought by the ABS and TPU phases, respectively. As such, the printed structure is stiff enough to enable self-supporting printing while being flexible to accommodate different face profiles to ensure a good fit and sealing. The polymeric mixtures are continuously stirred overnight using a roller mixer (DLAB Scientific, CA, USA). In the second step, both polymeric inks are mixed at a 2:1 ratio (ABS:TPU). The last step includes the addition of copper powders (10% (w/w)) to the dissolved polymer mixture. In order to guarantee the homogeneity of the suspension, the ink is mixed using a centrifugal mixer (AR-100, Thinky, CA, USA) for 2-3 minutes prior to loading the dispenser syringe.

Using a 3D extrusion printer (Hyrel Engine SR, Hyrel, USA), the ink is dispensed for a layer-by-layer deposition. This printing process is carried out while water is nebulized to the printing zone where the antiviral particle-dissolved polymer composite part is being printed, partially solidifying the polymer and entrapping the powder in the deposited filament due to the induced phase separation process.

The in-process solidification method is controlled to occur partially only for a balance of good fusion between two consecutively deposited layers and enough stiffness to sustain the following printed layers in air due to the existence of solidified polymer. For complete solidification throughout a printed part, it can be immersed, if needed, in a coagulation bath to fully remove the solvent. The collected solvent-relevant solution from the printing chamber and coagulation bath are post-processed to reclaim the solvent for its reuse, minimizing its environmental impact. After a drying cycle, an ABS-TPU-based 3D printed respirator is obtained.

1.11.2 Antibacterial testing

Staphylococcus aureus (ATCC 6358, ATCC, Manassas, Virginia, USA) cells were cultured in 1 mL of trypticase soy broth (TSB) (BD Difco, Becton, New Jersey, USA) in 24-well plates, along with the printed lattice-based ABS-TPU-copper disks of 8 mm in diameter. Additional positive controls (without disks) and negative controls (without *S. aureus* inoculum) were used. All groups were incubated at 37 °C, 150 rpm for 24 h. Each test run contained a single specimen, and it was run in triplicate. The antibacterial performance of the printed structures was determined using the Colony Forming Unit (CFU) counting method. For the testing of the reused disks, the same sample disks used previously were washed twice with phosphate-buffered saline (PBS, VWR, Radnor, Pennsylvania, USA), and once with 75% ethanol prior to the day of the test, then soaked in 75% ethanol for 30 min. Sterile disks were tested as previously described.

1.12 316L- hydroxyapatite EDS readings

Energy-dispersive spectroscopy (EDS, Hitachi S3000, Hitachi High-Technologies Co., Tokyo, Japan) was used to quantitively assess the composition of the metal-ceramic composite. As shown in Supplementary Fig. 18, the main elements of 316L are present (Fe, Cr, Mo, Mn, Ni, C) while the main elements of hydroxyapatite (Ca, P, O) are less present (~1.5-7.0%) due to the 316L-hydroxyapatite (HAp) ratio used (95-5% wt).

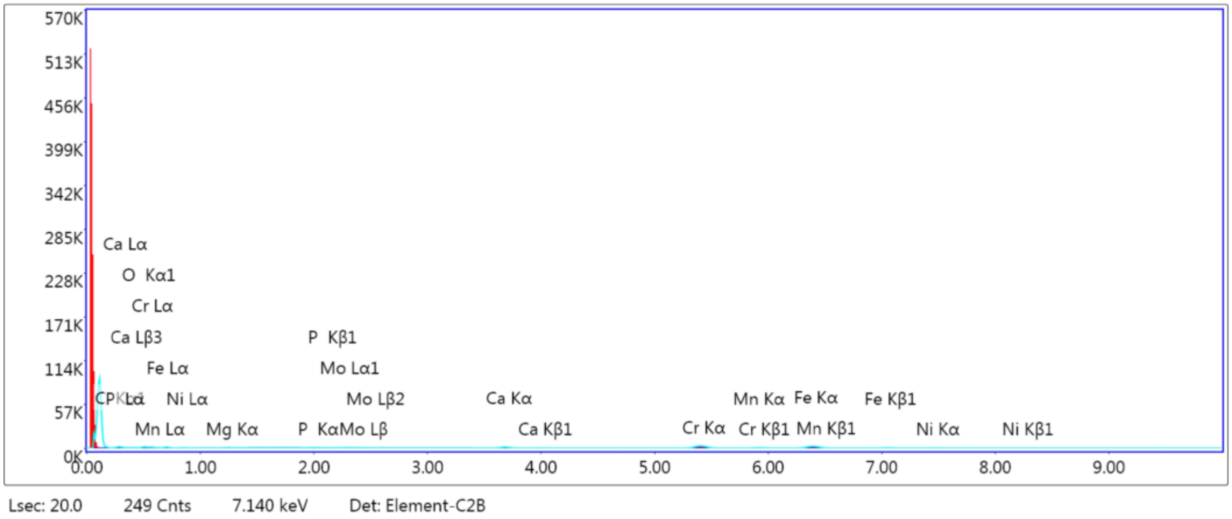

eZAF Smart Quant Results

Element	Weight %	Atomic %	Net Int.	Error %	Kratio	Z	A	F
C K	3.66	13.59	195.34	14.65	0.0127	1.3171	0.2640	1.0000
O K	1.93	5.39	227.54	11.71	0.0099	1.2604	0.4069	1.0000
MgK	1.46	2.68	160.60	11.56	0.0066	1.1645	0.3905	1.0018
P K	1.54	2.21	226.90	7.44	0.0127	1.1010	0.7446	1.0100
MoL	2.33	1.09	182.09	8.32	0.0188	0.8583	0.9322	1.0059
CaK	6.93	7.72	707.70	4.25	0.0765	1.0828	0.9575	1.0637
CrK	31.20	26.80	1901.82	3.37	0.3246	0.9719	0.9877	1.0837
MnK	2.25	1.83	108.38	11.33	0.0218	0.9512	0.9888	1.0315
FeK	42.22	33.76	1652.37	3.55	0.3980	0.9659	0.9644	1.0120
NiK	6.48	4.93	170.42	9.28	0.0607	0.9742	0.9524	1.0094

Supplementary Figure 18. EDS data for 316L-HAp composites.

1.13 Porous structure printing

For porous structure fabrication, sodium chloride powders (<177 µm, NaCl, GDF Chemicals, Powell, OH, USA) treated by an 80-mesh filter were mixed at 1:2 and 1:4 weight ratios, respectively, to 316L powders. The powder mixture was mixed with a 25% (w/v) ABS solution. The powder-to-binder ratios of approximately 55:45 by volume were used for all ink combinations. Particularly, the ABS solution and powders were mixed using a centrifugal mixer (AR-100, Thinky, CA, USA) for 2-3 minutes and then loaded in a disposable 5 mL syringe fitted with stainless-steel 18-23-gauge tips (Nordson EFD, Vilters, Switzerland) for printing.

The intra-filament pore size is determined by measuring the pore surfaces using ImageJ (NIH, USA) from various filament cross sections. The cross section of a printed filament is shown in Supplementary Fig. 19(a) for an exemplary purpose, showcasing that no NaCl particle is found after the water bath-based post-processing step. The pore distribution histogram is shown in Supplementary Fig. 19(b), where the mean and standard deviation of the sample population are 123.63 and 28.37 µm, respectively. Such measurements are in line with the original particle size as received (< 177 µm) after being filtered. The scattering of pore size is attributed to the discretization-based nature of the pore measurements. It is noted that higher concentrations of pores are observed in the inner-most region of the filament as a higher flow velocity is expected in that region, forcing the motion of the NaCl particles and resulting in a denser NaCl suspension[34].

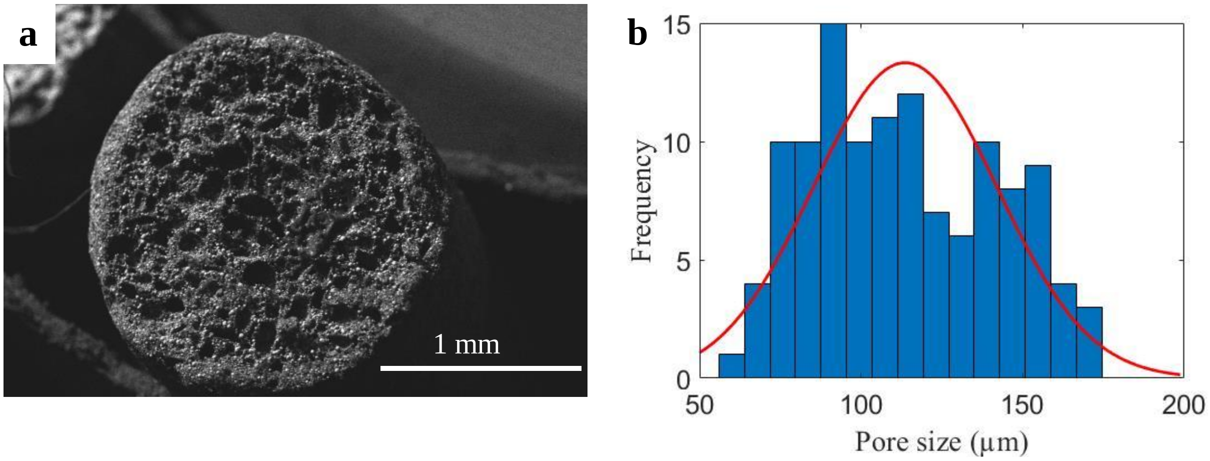

Supplementary Figure 19. Intra-filament porous structures. (**a**) Filament cross section details of green part. (**b**) Intra-filament pore size distribution.

1.14 Mechanical properties and fractometry of porous parts

Specimens of the identical geometry as specified in Supplementary Note 1.9.1 (mechanical properties of dense parts) were printed at different material porosity levels.

Dense parts (~92-100%, close to the theoretical density) present an ultimate tensile strength (UTS) of approximately 480 MPa, but the presence of pores detriments the mechanical properties due to the stress concentration on the boundaries surrounding the pores. Specifically, the increasing presence of porosity yields a decrease of UTS values down to 190, 120, and 95 MPa for porosities of 62, 52, and 42%, respectively.

From a fractometry perspective, the dense specimens present ductile trans-granular fracture as shown by the presence of dimples (Supplementary Fig. 20(a), red arrows), while the presence of high porosity level results in an unclear, non-obvious fracture mechanism[35] (Supplementary Fig. 20(b)).

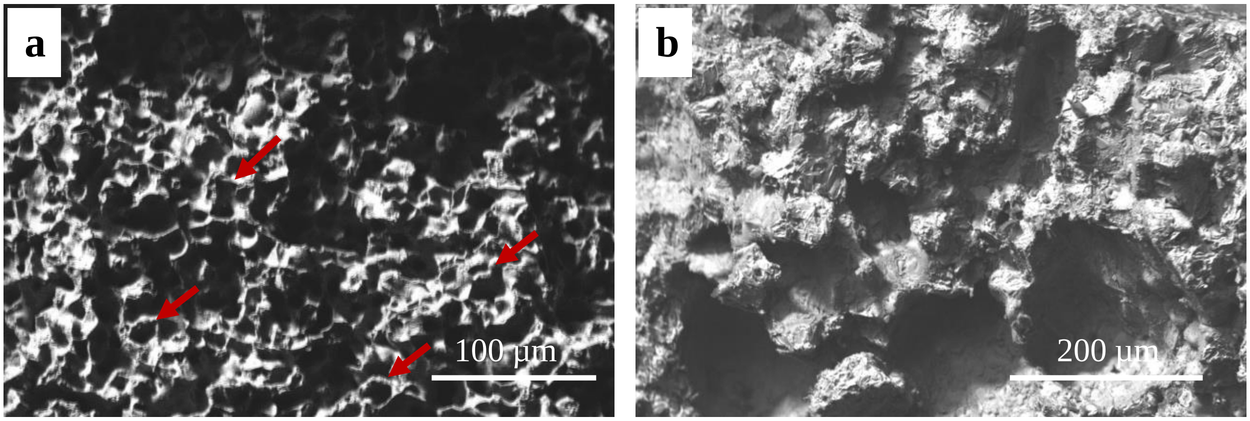

Supplementary Figure 20. Fracture surface morphology of (**a**) dense and (**b**) porous parts (52% porosity).

1.15 Cellular testing protocol and data

MC3T3-E1 subclone 4 pre-osteoblastic cells (ATCC, Rockville, MD) were cultured in α-minimal essential medium (α-MEM, Fisher Scientific, Pittsburgh, PA) supplemented with 10% fetal bovine serum (ATCC, Rockville, MD) and 1× antibiotic-antimycotic (Fisher Scientific, Pittsburgh, PA) in an incubator with 95% humidity and 5% CO_2 at 37°C. The complete medium was replaced every 2–3 days. When the cells were 80-90% confluent, the cells were harvested with TrypLE Express (Fisher Scientific, Pittsburgh, PA). Scaffolds with dense and porous filaments were sterilized with 70% ethanol for 30 minutes and washed with PBS (Fisher Scientific, Pittsburgh, PA) three times.

To study the cell proliferation and migration over the printed scaffolds, 150 µL MC3T3-E1 cells (4×10^5 cells/mL) were seeded on each disc scaffold in a 48-well plate and incubated for 30 min, and then 0.5 mL complete medium was gently added in each well. Then the samples were cultured in the incubator with 95% humidity and 5% CO_2 at 37 °C. The medium was changed every other day. The samples of the dense, mediumly porous, and highly porous discs were stained with a final concentration of 10 µg/mL Hoechst 33342 (Sigma-Aldrich, St. Louis, MO) to stain nuclei in blue and a final concentration of 10 µg/mL fluorescein diacetate (FDA) (Sigma-Aldrich, St. Louis, MO) to stain live cells in green on Days 1, 3, and 7, respectively. The images were captured using an EVOS FL inverted fluorescence microscope (Fisher Scientific, Waltham, MA).

The cell-fixing protocol used herein was the one previously used elsewhere[36]. Briefly, the samples were removed from the wells after culturing and rinsed using PBS three times. The samples were then immersed in a solution of 4% formaldehyde (Sigma Aldrich, St. Louis, MO, USA) in DI water for 25 min at 4 °C and 0.2% Triton X-100 (Sigma Aldrich, St. Louis, MI, USA) for 10 min to fix the cellular matter. The samples were further rinsed with an ethanol-DI water mixture with an increasing ethanol concentration (15%, 30%, 45%, 60%, 75%, 90%, and 100%, 10 min each). The samples were then air-dried using hexamethyldisilazane (HMDS, Sigma Aldrich, St. Louis, MO, USA). Finally, the cell-laden metallic samples were coated with Au-Pd for imaging using scanning electron microscopy (Hitachi S3000, Hitachi High-Technologies Co., Tokyo, Japan and Phenom XL, Thermo Fisher Scientific, Waltham, MA, USA).

1.16 Scaffold biocompatibility evaluation

As seen from the fluorescent images of Supplementary Fig. 21, the cell-seeded dense filament scaffolds are biocompatible with MC3T3-E1. It is noted that the cell density is visibly lower when compared to that of their porous counterparts.

a b c

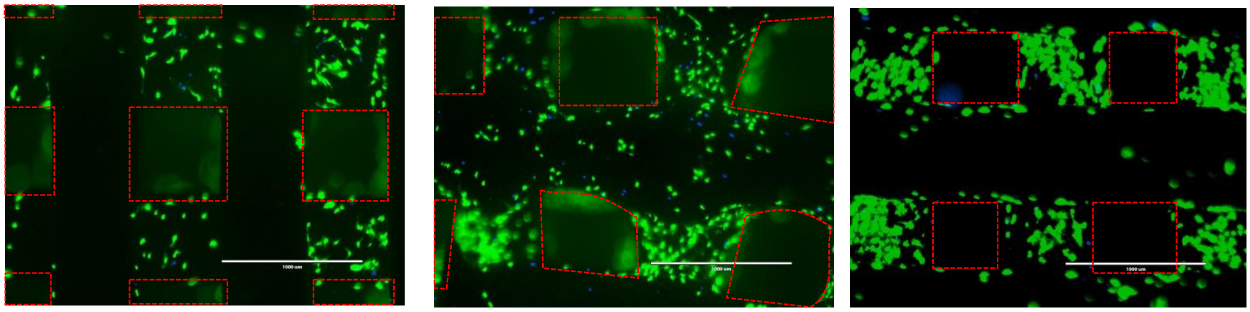

Supplementary Figure 21. Fluorescent images of cells on the surface of dense scaffolds after culturing for (**a**) 1, (**b**) 3, and (**c**) 7 days. Scale bars: 1 mm.

2. Supplementary References

[1]Hansen, C. M. 50 years with solubility parameters—past and future. *Progress in Organic Coatings* **51,** 77–84 (2004).

[2]Hansen, C. M. *Hansen Solubility Parameters a User's Handbook*. (CRC Press, Taylor & Francis Group, 2018).

[3]Peng, P., Shi, B., Jia, L. & Li, B. Relationship between Hansen solubility parameters of ABS and its homopolymer components of PAN, PB, and PS. *Journal of Macromolecular Science, Part B* **49,** 864–869 (2010).

[4]Flory, P. J. Thermodynamics of high polymer solutions. *The Journal of Chemical Physics* **10,** 51–61 (1942).

[5]Huggins, M. L. Some properties of solutions of long-chain compounds. *The Journal of Physical Chemistry* **46,** 151–158 (1942).

[6]Flory, P. J. *Principles of Polymer Chemistry*. (Cornell University Press, 1953).

[7]Yilmaz, L. & McHugh, A. J. Analysis of nonsolvent–solvent–polymer phase diagrams and their relevance to membrane formation modeling. *Journal of Applied Polymer Science* **31,** 997–1018 (1986).

[8]Tian, Y. *et al.* The investigation of Flory–Huggins interaction parameters for amorphous solid dispersion across the entire temperature and composition range. *Pharmaceutics* **11,** 420 (2019).

[9]Boom, R. M., van den Boomgaard, T., van den Berg, J. W. A. & Smolders, C. A. Linearized cloudpoint curve correlation for ternary systems consisting of one polymer, one solvent and one non-solvent. *Polymer* **34,** 2348–2356 (1993).

[10]Xu, L. & Qiu, F. Simultaneous determination of three Flory–Huggins interaction parameters in polymer/solvent/nonsolvent systems by viscosity and cloud point measurements. *Polymer* **55,** 6795–6802 (2014).

[11]Dong, R., Zhao, J., Zhang, Y. & Pan, D. Morphology control of polyacrylonitrile (PAN) fibers by phase separation technique. *Journal of Polymer Science Part B: Polymer Physics* **47,** 261–275 (2008).

[12]Vandezande, P., Gevers, L. E. & Vankelecom, I. F. Solvent resistant nanofiltration: Separating on a molecular level. *Chem. Soc. Rev.* **37,** 365–405 (2008).

[13]Kharraz, J. A., Bilad, M. R. & Arafat, H. A. Flux stabilization in membrane distillation desalination of seawater and brine using corrugated PVDF membranes. *Journal of Membrane Science* **495,** 404–414 (2015).

[14]Mazinani, S., Darvishmanesh, S., Ehsanzadeh, A. & Van der Bruggen, B. Phase separation analysis of Extem/solvent/non-solvent systems and relation with membrane morphology. *Journal of Membrane Science* **526,** 301–314 (2017).

[15]Wei, Y.-M., Xu, Z.-L., Yang, X.-T. & Liu, H.-L. Mathematical calculation of binodal curves of a polymer/solvent/nonsolvent system in the phase inversion process. *Desalination* **192,** 91–104 (2006).

[16]Strathmann, H., Kock, K., Amar, P. & Baker, R. W. The formation mechanism of asymmetric membranes. *Desalination* **16,** 179–203 (1975).

[17]Su, A. C. & Fried, J. R. Phase behavior of ternary polymer blends. *Polymer Engineering and Science* **27,** 1657–1661 (1987).

[18]Aryanti, P. T., Ariono, D., Hakim, A. N. & Wenten, I. G. Flory-Huggins based model to determine thermodynamic property of polymeric membrane solution. *Journal of Physics: Conference Series* **1090,** 012074 (2018).

[19]Soltan, N., Ning, L., Mohabatpour, F., Papagerakis, P. & Chen, X. Printability and cell viability in bioprinting alginate dialdehyde-gelatin scaffolds. *ACS Biomaterials Science & Engineering* **5,** 2976–2987 (2019).
[20]Venault, A., Chang, Y., Wang, D.-M. & Bouyer, D. A review on polymeric membranes and hydrogels prepared by vapor-induced phase separation process. *Polymer Reviews* **53,** 568–626 (2013).
[21]Su, Y. S. *et al.* Interplay of mass transfer, phase separation, and membrane morphology in vapor-induced phase separation. *Journal of Membrane Science* **338,** 17–28 (2009).
[22]Stropnik, Č., Musil, V. & Brumen, M. Polymeric membrane formation by wet-phase separation; turbidity and shrinkage phenomena as evidence for the Elementary Processes. *Polymer* **41,** 9227–9237 (2000).
[23]Chang, H.-Y. & Venault, A. Adjusting the morphology of poly(vinylidene fluoride-co-hexafluoropropylene) membranes by the VIPS process for efficient oil-rich emulsion separation. *Journal of Membrane Science* **581,** 178–194 (2019).
[24]Broens, L., Altena, F. W., Smolders, C. A. & Koenhen, D. M. Asymmetric membrane structures as a result of phase separation phenomena. *Desalination* **32,** 33–45 (1980).
[25]Xue, T. J., McKinney, M. A. & Wilkie, C. A. The thermal degradation of polyacrylonitrile. *Polymer Degradation and Stability* **58,** 193–202 (1997).
[26]Hanna, S. B., Yehia, A. A., Ismail, M. N. & Khalaf, A. I. Preparation and characterization of carbon fibers from polyacrylonitrile precursors. *Journal of Applied Polymer Science* **123,** 2074–2083 (2011).
[27]Klar, E. & Samal, P. K. *Powder metallurgy stainless steels processing, microstructures, and properties*. (ASM International, 2007).
[28]Kováčik, J. The tensile behaviour of porous metals made by Gasar process. *Acta Materialia* **46,** 5413–5422 (1998).
[29]Röttger, A. *et al.* Microstructure and mechanical properties of 316l austenitic stainless steel processed by different SLM devices. *The International Journal of Advanced Manufacturing Technology* **108,** 769–783 (2020).
[30]Mao, C.-X., Khalily, M., Xiao, P., Brown, T. W. & Gao, S. Planar sub-millimeter-wave array antenna with enhanced gain and reduced sidelobes for 5G broadcast applications. *IEEE Transactions on Antennas and Propagation* **67,** 160–168 (2019).
[31]Rao, P. R. *Communication Systems*. (McGraw Hill Education (India), 2013).
[32]Pozar, D. M. *Microwave Engineering*. (Wiley, 2012).
[33]Teshirogi, T. & Yoneyama, T. *Modern Millimeter-wave Technologies*. (IOS Press, 2001).
[34]Lecampion, B. & Garagash, D. I. Confined flow of suspensions modelled by a frictional rheology. *Journal of Fluid Mechanics* **759,** 197–235 (2014).
[35]Dudrova, E. & Kabátova, M. A review of failure of sintered steels: Fractography of static and dynamic crack nucleation, coalescence, growth and propagation. *Powder Metallurgy* **59,** 148–167 (2016).
[36]Ren, B. *et al.* Improved osseointegration of 3D printed Ti-6Al-4V implant with a hierarchical micro/nano surface topography: An in vitro and in vivo study. *Materials Science and Engineering: C* **118,** 111505 (2021).
