# Supplementary material for: Vapor-induced phase-separation-enabled versatile direct ink writing
Source: Nat Commun. 2024 Apr 9;15:3058. doi: 10.1038/s41467-024-47452-9 (PMC11003993; doi:10.1038/s41467-024-47452-9)
Supplement: Supplementary file 3 — Description of Additional Supplementary Files [file 41467_2024_47452_MOESM3_ESM.pdf]

Description of Additional Supplementary Files

Supplementary Movie Legends:

Supplementary Movie 1:
Title: Printing process
Description: Printing in air under VIPS

Supplementary Movie 2: Water leakage testing of a printed part
Title: Leakage testing
Description: Water leakage testing of a printed part
